# The Contribution of Echocardiography to the Diagnosis and Prognosis Stratification of Diabetic Cardiomyopathy

**DOI:** 10.3390/diagnostics15202587

**Published:** 2025-10-14

**Authors:** Maria Ioannou, Dimitrios Karelas, Alkistis Eleni Kalesi, Georgios Parpas, Christos A. Papanastasiou, Constantinos H. Papadopoulos, Angeliki Mouzarou, Nikolaos P. E. Kadoglou

**Affiliations:** 1 Department of Cardiology, Limassol General Hospital, State Health Services Organization, 4131 Limassol, Cyprus; mar.ioannou25@gmail.com; 2 Department of Cardiology, Red Cross General Hospital of Athens, 11526 Athens, Greece; dim.f.karelas@gmail.com (D.K.); papcost@gmail.com (C.H.P.); 3 Department of Cardiology, Tzaneio General Hospital of Piraeus, 18532 Piraeus, Greece; eileen_calessis@yahoo.gr; 4 Department of Internal Medicine, Nicosia General Hospital, State Health Services Organization, 2029 Nicosia, Cyprus; parpas.giorgos@outlook.com; 5 Department of Cardiology, 424 Military Hospital, 56429 Thessaloniki, Greece; cpapanas@gmail.com; 6 Department of Cardiology, Paphos General Hospital, State Health Services Organization, 8026 Paphos, Cyprus; a.mouzarou@shso.org.cy; 7 Medical School, University of Cyprus, 1678 Nicosia, Cyprus

**Keywords:** diabetic cardiomyopathy, echocardiography, diagnosis

## Abstract

The relationship of diabetes mellitus (DM) with cardiovascular mortality and morbidity has been widely established. Diabetic cardiomyopathy (DBCM) has been increasingly recognized as the development of cardiac dysfunction accompanied by heart failure (HF) symptoms in the absence of obvious causes like coronary artery disease (CAD), hypertension (HTN) or valvular diseases. The objective of this review is to critically appraise the role of echocardiography in the diagnosis and prognostic stratification of DBCM. Echocardiography remains the first-line imaging modality due to its availability, repeatability, non-invasive nature and ability to assess structural and functional changes. Classical echocardiographic indices such as left ventricular hypertrophy and systolic and diastolic dysfunction assessment provide valuable information but they lack sensitivity, often remaining normal until advanced stages of DBCM. Recently developed echocardiographic modalities, including strain imaging, myocardial work indices and left atrial strain, may allow for earlier detection of subclinical myocardial dysfunction, having important prognostic implications. However, these advanced modalities require high imaging quality, expertise and standardization, being subject to technical and physio-logical limitations. Stress echocardiography, particularly exercise-based protocols, is an increasingly recognized, valuable tool for unmasking exertional abnormalities in filling pressures, myocardial reserve and pulmonary pressures that are not evident at rest. Until now, stress echocardiography requires validation in large cohorts to assess its prognostic power. This review highlights the importance of timely recognition of DBCM, underscores the advantages and disadvantages of current echocardiographic approaches and outlines future perspectives in multimodality imaging to improve patient outcomes.

## 1. Introduction

Diabetic cardiomyopathy (DBCM) was first described in 1972 by Rubler et al. [1], who identified the development of myocardial dysfunction in diabetic patients in the absence of other underlying causes such as coronary artery disease (CAD), hypertension (HTN), or valvular disease [2]. Although DBCM is now considered a distinct condition, it is still underrepresented in current clinical practice. Most large trials and guidelines continue to consider it as a pathological condition within the clinical syndrome of heart failure with preserved ejection fraction (HFpEF), rather than as an independent entity. This lack of focused research investigation has resulted in significant gaps in knowledge, particularly regarding its pathophysiology, prompt diagnosis, clinical progress, and optimal management [3].

DBCM is the result of hyperglycemia-driven metabolic alterations, oxidative stress, chronic inflammation, and mitochondrial dysfunction [2,4,5]. These mechanisms result in a sequence of events, including interstitial fibrosis, myocyte apoptosis, and impaired calcium homeostasis that lead to cardiac remodeling, initially manifesting as diastolic and progressively evolving to systolic dysfunction. Unlike other cardiomyopathies, the subtle progression of DBCM lasts for years before it evolves to HF with reduced ejection fraction (HFrEF), underscoring the need for early detection and timely treatment.

Although DBCM has been described in both type 1 (T1DM) and type 2 (T2DM) diabetes mellitus, most studies have enrolled patients with T2DM, causing molecular alterations and impaired cardiac function. On the contrary, the presence and characterization of DBCM in T1DM remains controversial. While systemic metabolic derangements such as hyperglycemia occur in both types, the impact of insulin on the underlying mechanisms may differ, regarding insulin resistance with hyperinsulinemia in T2DM versus insulin deficiency in T1DM. This divergency in pathophysiology has not been studied and how it may influence the evolution to DBCM between those types of DM [6].

Echocardiography plays a crucial role in the assessment of myocardial involvement in diabetic subjects as it is a non-invasive, repeatable, and easily accessible diagnostic tool. However, conventional echocardiographic parameters such as left ventricular (LV) ejection fraction (LVEF) often remain normal until the advanced stages of the disease. This limitation has led to increasing interest in novel, more sensitive echocardiographic techniques that can reveal subtle myocardial dysfunction before the development of overt HF [7]. Classical indices used to assess the presence of LV hypertrophy, diastolic, and systolic dysfunction offer essential insights into structural remodeling and functional impairment [7,8]. However, the above often fail to detect the subtle myocardial changes that characterize the early, preclinical stages of DBCM. To overcome this limitation, advanced echocardiographic techniques have emerged, providing a more accurate evaluation of myocardial mechanics. Among these, global longitudinal strain (GLS) has proven especially valuable, revealing subclinical systolic dysfunction even when LVEF appears preserved [9,10]. Complementary tools such as myocardial work indices (MWI) incorporate loading conditions into the assessment of contractile performance. Myocardial work (MW) is an evolving echocardiographic tool linked with the pathophysiology of myocardial function, but more studies are required to clarify its clinical impact and its advantages over speckle tracking analysis (STA) and to set its limitations [11,12]. Exercise stress echocardiography (ESE) and its specific protocol diastolic stress echocardiography (DSTE), allows the detection of functional abnormalities such as impaired diastolic relaxation, reduced contractile reserve, and elevated filling pressures that are not evident at rest [13,14]. Since the last decade, it has been proposed as an essential modality for HFpEF diagnosis in cases of patients with intermediate probability of HFpEF. The latter cohort represents a growing number of individuals with HF symptoms, but without strong evidence for HFpEF in resting echocardiography. DSTE may add value in HFpEF diagnosis. However, its role in DBCM diagnosis and prognosis remains to be proved.

Unlike broader HFpEF reviews, this review focuses on DBCM and highlights its unique imaging challenges. In the first part of our review, the main pathophysiological mechanisms leading to the development of DBCM are described, and metabolic disturbances at cellular and molecular levels that are related to clinical phenotypes are outlined. In the second part, a comprehensive review of all available echocardiographic modalities and their value in DBCM diagnosis is attempted, analyzing not only the conventional echocardiographic indices, but also novel echocardiographic parameters which have been tested in clinical practice. By critically appraising current echocardiographic techniques within the specific context of DBCM, this review aims to bridge the gap between evolving pathophysiological insights and diagnostic strategies.

## 2. Pathophysiology of Diabetic Cardiomyopathy

DBCM represents a distinct cardiac complication of DM, characterized primarily by diastolic dysfunction and often evolving to systolic dysfunction. These impairments arise from structural and molecular changes, including cardiomyocyte hypertrophy, interstitial fibrosis, enhanced inflammatory cell infiltration, oxidative stress and increased apoptosis [8,10]. As such, the pathophysiology of DBCM is complex and multifactorial, reflecting the interaction of metabolic, cellular and neurohormonal pathways that progressively undermine heart structure and function.

At the center of this process are chronic hyperglycemia and insulin resistance. The former is not only a marker of poor metabolic control but also a driver for various processes resulting in tissue damage [3]. In hyperglycemic condition an amount of excessive glucose levels divert into alternative metabolic pathways such as the polyol pathway and the hexosamine biosynthetic pathway. The latter leads to prolonged O-GlcNAcylation, which disrupts calcium homeostasis, impairs mitochondrial function, promotes apoptosis and weakens insulin signaling [9]. Additionally, sustained hyperglycemia promotes the formation of advanced glycation end-products (AGEs) and activates protein kinase C (PKC). Both promote oxidative stress and inflammation [13]. Besides this, AGEs, through non-enzymatic glycation, form irreversible cross-links between adjacent collagen fibers, a process that increases myocardial fibrosis and stiffness and impairs LV relaxation, a key characteristic of diastolic dysfunction in DBCM. Furthermore, the interaction of AGEs with their receptors (RAGEs) further promotes inflammation by activating transcription factors like the Nuclear Factor Kappa B (NF-kB) [3].

Over time, this glucose overload causes mitochondrial dysfunction, oxidative stress through higher production of reactive oxygen species (ROS) and promotes energy imbalance [15]. The frequent co-existence of insulin resistance causes depletion of glucose transporter-4 (GLUT-4), along with increased lipolysis in adipose tissue, resulting in reduced glucose and increased fatty acid (FA) uptake by cardiomyocyte. Thus, the myocardial metabolism shifts toward FA oxidation. The resulting mismatch between fuel supply (e.g., FAs) and oxidative capacity leads to the accumulation of ceramides and diacylglycerols which further impair insulin signaling and mitochondrial function. This process, usually described as lipotoxicity, along with glucose overload and mitochondrial dysfunction that ensues, renders the diabetic heart vulnerable during ischemic stress [16].

Importantly, insulin resistance also dampens AMP-activated protein kinase activity, a key regulator of energy balance and autophagy. This suppression of autophagy (mitophagy, glycophagy) leads to accumulation of dysfunctional mitochondria, misfolded proteins, and glycogen, further compromising cardiomyocyte survival by promoting cardiomyocyte apoptosis and loss of contractile units [16,17].

Metabolic stress in diabetes triggers chronic low-grade inflammation through activation of pathways such as NLRP3 inflammasome and NF-κB leading to cytokine release (IL-1β, IL-6, TNF-α) [18,19]. At the same time hyperglycemia and insulin resistance impair renin–angiotensin–aldosterone system (RAAS) balance by enhancing Angiotensin II signaling and suppressing the protective ACE2/Ang-(1-7)/Mas axis [20]. These processes collectively drive fibroblast activation, collagen deposition, oxidative stress and endothelial dysfunction ultimately promoting hypertrophy and fibrosis, key features of DBCM [21].

As these metabolic insults accumulate, they promote inflammatory signaling. Activation of NLRP3 inflammasome and the NF-kB pathway promotes the release of pro-inflammatory cytokines such as IL-1β, IL-6, and TNF-α, establishing a low-grade inflammation state [18,19]. Concurrently, the renin–angiotensin–aldosterone system (RAAS) becomes overactivated. Notably, hyperglycemia was shown to promote intracellular Angiotensin II production in cardiomyocytes independently of circulating RAAS. In addition, insulin resistance may downregulate the protective ACE2/Ang-(1-7)/Mas receptor axis, thus allowing the classical RAAS arm to dominate [20]. Together, they promote fibroblast proliferation, collagen deposition, oxidative stress, and endothelial dysfunction, contributing to hypertrophy and fibrosis, distinct features of DBCM [21].

Microvascular dysfunction is also an important aspect of DBCM with HF with preserved ejection fraction (HFpEF) phenotype. Diabetes-associated endothelial dysfunction, characterized by reduced nitric oxide (NO) bioavailability and endothelial nitric oxide synthase (eNOS) uncoupling, impairs myocardial perfusion and oxygen delivery [22]. Together with endothelin-1 (ET1) signaling and AGE-RAGE activation, they promote vascular stiffening, interstitial fibrosis and cardiomyocyte hypertrophy. The resultant capillary rarefaction and reduced metabolic reserve further compromise myocardial structure and function [8,21].

Another defining feature of diastolic and systolic impairment in DBCM is abnormal calcium handling. Reduced L-type calcium channel activity diminishes calcium influx, while oxidative modification and hyperphosphorylation of ryanodine receptors (RyR2) promote calcium leak from the sarcoplasmic reticulum. Concurrently, downregulation of sarcoplasmic/endoplasmic reticulum Ca^2+^-ATPase 2a (SERCA2a), hampers calcium reuptake, prolonging diastolic relaxation and contributing to stiffness and arrhythmogenesis. The net effect is impaired relaxation, reduced contractility, and a predisposition to heart failure and arrhythmias [23].

Beyond the well-established mechanisms, emerging evidence has highlighted the role of microRNAs (miRNAs) in the pathogenesis of DBCM. These small non-coding RNAs primarily regulate gene expression post-transcriptionally and they are increasingly recognized as modulators of both protective and pathogenic processes in the diabetic heart [23,24,25].

In addition to the above, clinical comorbidities can significantly modify the cardiac phenotype. HTN is a common comorbidity in diabetes, especially among T2DM, which can per se lead in myocardial remodeling and dysfunction. While the current review emphasizes diabetic cardiomyopathy as the net result of diabetes-related mechanisms, in the real-world HTN often coexists. Hence, both entities cause overlapping structural and functional changes. This overlap highlights the challenge to carefully distinguish the impact of diabetes from HTN on cardiac function. Nevertheless, our findings predominantly describe the impact of isolated diabetes on cardiac function. We focused entirely on diabetic cohorts and excluded studies where diabetic patients consisted of a percentage of the population. Moreover, we selected studies where diabetes was clearly stated as the cause of cardiac dysfunction.

In summary, DBCM is a consequence of the interplay between chronic hyperglycemia and insulin resistance, which trigger a constellation of pathological processes involving metabolic derangement mitochondrial dysfunction, inflammation, oxidative stress, and impaired calcium homeostasis. These changes lead to structural remodeling and progressive impairment of both systolic and diastolic function.

## 3. Classical Echocardiographic Indices for Diagnosis and Prognosis

Conventional echocardiography serves as a primary imaging modality for accurate evaluation of cardiac anatomy and overall cardiac function, especially the diastolic one. Classical echocardiographic indices, combining two-dimensional and Doppler techniques, play a pivotal role in the diagnosis and prognosis of DBCM and include:

### 3.1. Left Ventricular Hypertrophy (LVH)

DM is characterized by insulin resistance and subsequent hyperglycaemia, which stimulate pro-hypertrophic changes in the myocardium, reactive interstitial fibrosis, and extracellular collagen deposition [26]. LVH is considered the most common echocardiographic finding in individuals with type 2 DM. Numerous studies have confirmed the strong association between diabetes and LVH, with evidence extending to individuals in the prediabetic stage, particularly those with impaired glucose tolerance (IGT) [26,27,28,29,30]. The Strong Heart Study identified independent associations between IGT and increased LV relative wall thickness (RWT), as well as a higher LV mass-to-height ratio. The Cardiovascular Health Study found that, in a cohort of 5201 men and women, the ventricular septal and left posterior myocardial wall thickness was greater in diabetic versus nondiabetic individuals [28]. These findings indicate that structural remodeling of the heart begins early, even before the onset of clinical DM [29].

Notably, an old postmortem study identified LVH as the earliest feature of DBCM [1]. Those pathophysiological characteristics were confirmed later on by histopathological studies in DBCM, showing alterations in myocardial structure with prominent LVH, myocardial enlargement and fibrosis [31]. A recent long-term study has highlighted a relationship between DBCM and left ventricular mass index (LVMi), between young and mid-adulthood. It demonstrated a progressive wall thickening along with elevated glucose levels [32]. Unambiguously, LVH significantly impacts prognosis [32,33] but appears to be partially reversible with tight blood glucose control [34,35]. This underscores the importance of close collaboration between diabetologists and cardiologists since hyperglycaemia, even in the prediabetic stage, may drive LVH and adverse cardiac remodeling.

### 3.2. Diastolic Dysfunction

The histopathologically observed changes in patients with DM, such as myocardial hypertrophy and interstitial fibrosis, may ultimately contribute to LV stiffening [36]. Those changes lead to high LV filling pressure (LVFP), the cornerstone of diastolic dysfunction and a key characteristic of DBCM [37]. Broad literature evidence supports the very common finding of diastolic dysfunction among type 2 diabetic patients (T2DM) [37,38,39]. A recent study found that 61% of asymptomatic patients with newly diagnosed DM (within one year) already exhibited LV diastolic dysfunction [40]. Similar findings were observed in individuals with IGT, but not in those with impaired fasting glucose (IFG) [41]. Despite several potential confounders, the association of DM with diastolic dysfunction is also reported in studies comparing diabetic vs. non-diabetic controls matched for age, sex, weight, and systolic blood pressure [42].

The key echocardiographic parameters of diastolic dysfunction include the transmitral E/A ratio, E/e’ ratio, early diastolic velocity (e’) using tissue Doppler imaging (TDI), left atrial (LA) volume index (LAVi), and the assessment of tricuspid regurgitation (TR) using Doppler ultrasound. The duration of uncontrolled DM correlates positively with the incidence and severity of diastolic dysfunction [40]. Jain et al. demonstrated a strong association between elevated hemoglobin A1C (HbA1c) levels and diastolic dysfunction. In their study, half of the participants with HbA1c >9.5% exhibited a restrictive filling pattern [43]. However, most studies report that DM patients with diastolic dysfunction present with impaired relaxation or pseudonormal filling patterns [38].

Initial studies showed decreased transmitral E/A ratio in patients with DM, even when LV mass remains normal [44]. The E/e’ ratio, which reflects LVFP, was found significantly elevated in patients with HbA1c levels ≥8.1% [40]. In agreement to previous studies, From et al. observed an independent association of DM duration of over four years with E/e’ > 15 [45]. Importantly, a septal E/e’ ratio >15 in diabetic patients is associated with the subsequent development of HF and increased mortality independent of CAD, HTN, or other echocardiographic parameters [46].

Current evidence suggests that LA dilatation may serve as a marker of diastolic dysfunction in diabetic patients [47]. The CARDIA (Coronary Artery Risk Development in Young Adults) study demonstrated that over a 5-year period, diabetes was not associated with either unindexed LA diameter or LA diameter indexed to body surface area (BSA) or height. However, after a 20-year follow-up period, a significant association emerged between diabetes and increased unindexed and indexed LA diameters [48]. Similarly, the TODAY (Treatment Options for Type 2 Diabetes in Adolescents and Youth) trial, reported that LA diameter, even when indexed for BSA or height, showed no correlation with poorly controlled DM [49]. These findings raise concerns about the utility of LA diameters as a reliable marker of DBCM [50].

The use of LA volumes has proven more effective in detecting subclinical cardiac damage in DM patients. Over the past decade, previous studies in DM patients have confirmed common LA enlargement, as measured by LA volumes and indexes [46,50,51,52]. Recent evidence highlights a strong association between disease duration and both increased LA volume and impaired LA function [50]. These parameters constitute independent predictors of cardiovascular events in diabetic patients [46,49,51,53,54]. Notably, an increased LAVi ≥ 32 mL/m^2^ has been identified as an independent and incremental predictor of cardiovascular morbidity and mortality in diabetic patients without known cardiovascular disease [55].

**Figure 1:**
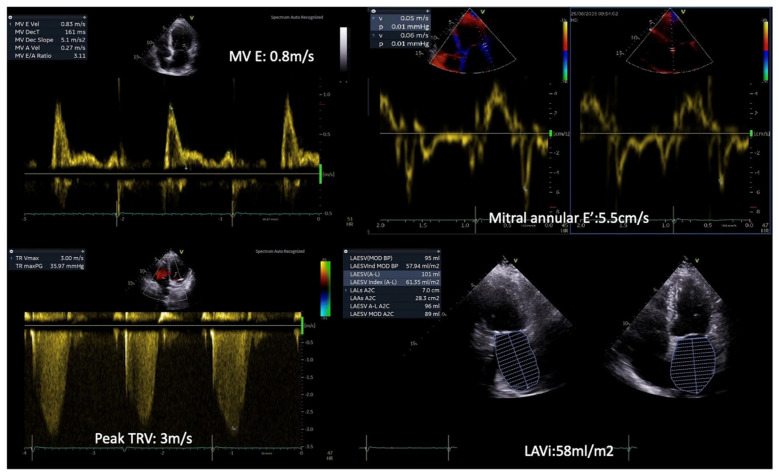
Patient with T2DM and exertional dyspnea. Rest echocardiography revealed diastolic dysfunction grade III, with an MV E/E’ ratio = 15, a Peak TR velocity: 3 m/s and severe left atrial dilatation (LAVi: 58 mL/m^2^). (Original images from authors’ institutions).

Diastolic dysfunction in individuals with DM is associated with a worse prognosis, as these patients face a higher risk of developing HF, compared to their counterparts without diastolic dysfunction [45]. Optimizing DM treatment and reducing HbA1c levels may lead to improvements in diastolic dysfunction, potentially delaying the progression to HF [56] (Figure 1).

### 3.3. LV Systolic Dysfunction

Systolic dysfunction is a later manifestation, usually occurring after diastolic dysfunction in diabetic patients. DBCM, in its early stages, includes a latent subclinical period characterized by structural and functional abnormalities, including LVH, fibrosis, stiffness, and cell-signaling disruptions. These pathophysiological changes often progress to systolic dysfunction and eventually lead to a clinical HF [3,57]. Subtle systolic dysfunction is often not detected using standard two-dimensional echocardiography techniques [58]. Advanced imaging modalities, such as TDI, speckle-tracking echocardiography (STE), and GLS assessment, are more sensitive and better suited for identifying early impairments in systolic function. In DM patients with normal conventional parameters [LVEF, fractional shortening (FS)], it appeared that they present a significant decrease in both longitudinal and radial systolic strain values by STE [59]. More recent studies showed that subclinical myocardial dysfunction with a progressive decline in GLS was observed during follow-up using 2D and 3D-STE, independent of other cardiovascular risk factors, while no significant changes were noted in global circumferential or radial strain [60]. Ceyhan et al. showed that there is systolic and diastolic LV dysfunction even in prediabetic patients, as in patients with diabetes, by using the longitudinal peak systolic strain and the peak systolic and diastolic strain rates [61]. Finally, Leung et al. reported possible improvement in GLS after optimization of treatment and reduction in HbA1c [56].

**Figure 2:**
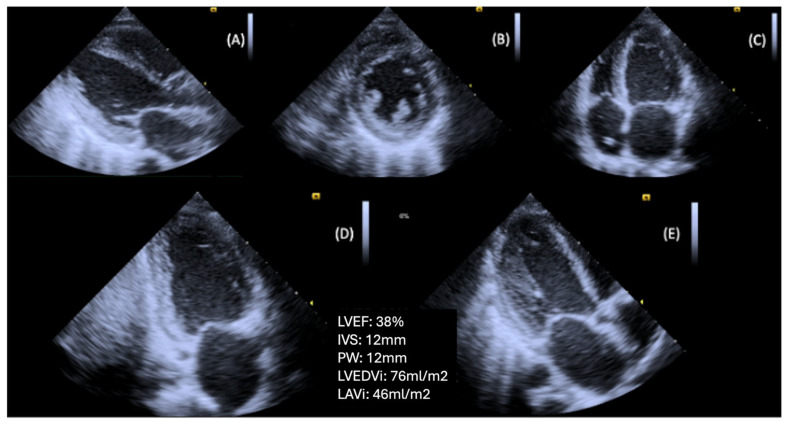
Patient with T2DM and advanced DBCM, causing left ventricular hypertrophy and systolic dysfunction. (**A**) Parasternal long axis view, (**B**) Parasternal short axis view (level of papillary muscles), (**C**) Apical four-chamber view, (**D**) Apical two-chamber view, (**E**) Apical long axis view (Original images from authors’ institutions).

Prolonged cumulative exposure to uncontrolled DM from early adulthood to middle age is a risk factor for adverse LV remodeling and subclinical LV dysfunction, initially diastolic and eventually systolic. The duration of DM may predict clinical HF, including both preserved and reduced LVEF, later in life [36]. A 1% increase in HbA1c is associated with an 8% higher risk of HF [62]. Presumably, the early commitment to intensive glucose lowering can slow the progression of DBCM and reduce the risk of developing clinical HF (Figure 2).

### 3.4. Right Ventricular Systolic and Diastolic Dysfunction

Similarly to LV, subclinical dysfunction of the right ventricle (RV) was found in diabetic patients [63]. DBCM involves RV, as evidenced by RV remodeling, systolic and diastolic dysfunction among men with uncontrolled type 2 diabetes, mirroring the alterations observed in LV structure and function. These observations suggest that RV impairment may be a component of the DBCM phenotype [64].

Current evidence supports the use of indices of RV function, such as peak myocardial systolic velocity (S’), early diastolic myocardial velocity (e’), and late diastolic myocardial velocity (a’), as measured by TDI. These indices were found to be significantly lower in diabetic patients compared to the control group [65]. A cross-sectional observational study comparing RV dysfunction in diabetic and non-diabetic subjects using echocardiography revealed significant differences in RV end-diastolic diameter (RVEDD) and E/e’, as well as a significantly higher tricuspid peak late diastolic flow (A) velocity in diabetic patients. The same study also found that Tricuspid Annular Plane Systolic Excursion (TAPSE) and E/A ratio had a significant correlation with the duration of DM. Additionally, RV e’ showed a significant correlation with HbA1c [66]. The Maastricht Study published in 2020 concluded that prediabetes is associated with structural and functional changes in the right atria and ventricle due to the direct myocardial impact of hyperglycaemia [67]. Therefore, even at the preclinical stage, echocardiography can be a powerful tool to identify this RV dysfunction. The clinical impact of current anti-diabetic therapy on RV function remains to be proved.

Despite their clinical value, conventional echocardiographic indices have several limitations in the context of diabetic cardiomyopathy. Left ventricular hypertrophy, diastolic dysfunction and ejection fraction measurements are influenced by loading conditions and heart rate, which may confound the interpretation of isolated effects. Furthermore, conventional indices often lack the sensitivity to detect subclinical myo-cardial dysfunction, as LVEF and diastolic parameters may remain within normal ranges until advanced stages of the disease delaying the diagnosis.

## 4. Novel Echocardiographic Indices for Diagnosis and Prognosis

### 4.1. Speckle Tracking Echocardiography of the LV

**Figure 3:**
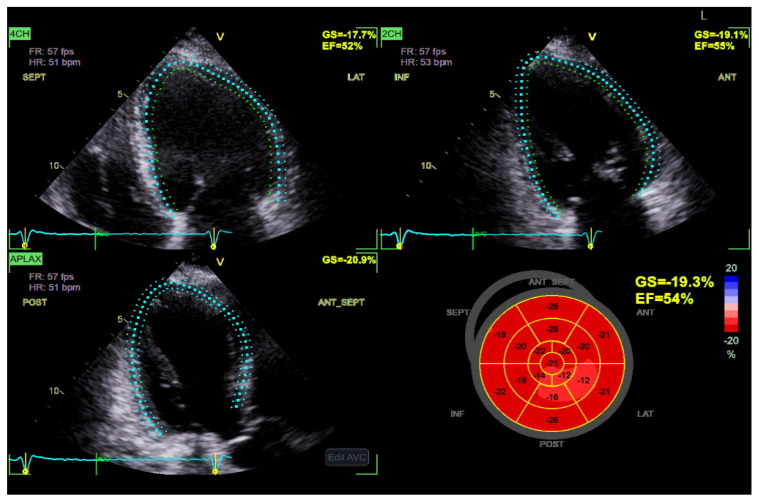
Global longitudinal strain (GLS) has increased sensitivity in diagnosing DBCM at its early stages and could be a useful index for the follow-up in patients with T2DM. A progressive decrease in GLS, in serial echocardiographic studies of the same patient, may serve as an early marker of emerging diabetic cardiomyopathy. (Original images from authors’ institutions).

STE has emerged as a valuable imaging modality in detecting subclinical myocardial dysfunction in DBCM [68]. Traditional echocardiographic parameters, such as LVEF, often remain within normal limits in the early stages of DBCM, limiting their utility in identifying subtle myocardial abnormalities [69]. In contrast, STE enables the quantification of myocardial deformation by assessing global and segmental strain patterns, thereby offering superior sensitivity in detecting early systolic and diastolic dysfunction [69,70] (Figure 3). Among the various strain parameters, GLS has been extensively validated as a robust marker of myocardial dysfunction. Several studies have demonstrated that diabetic patients exhibit significantly reduced absolute GLS values compared to non-diabetic controls, even when LVEF is preserved [6,71]. A GLS value above −18% is considered indicative of early myocardial impairment, correlating with both metabolic dysregulation and cardiovascular risk [72]. Furthermore, longitudinal strain impairment has been shown to precede changes in circumferential or radial strain, highlighting the vulnerability of longitudinal subendocardial fibers to diabetic metabolic disturbances, fibrosis, and microvascular dysfunction [72,73].

Importantly, GLS also holds significant prognostic value in patients with DBCM. Reduced absolute values of GLS have been independently associated with adverse cardiovascular outcomes, including HF hospitalization, major adverse cardiac events (MACE), and all-cause mortality, even in diabetic individuals with preserved LVEF [74,75]. A longitudinal study by Ersbøll et al. demonstrated that impaired GLS was a stronger predictor of cardiovascular mortality than LVEF in patients with diabetes and established heart disease [76]. Moreover, in asymptomatic individuals with type 2 diabetes mellitus and normal LVEF, subclinical LV dysfunction identified by impaired GLS was shown to be an independent predictor of all-cause mortality and hospitalization over nearly a decade of follow-up [77]. Complementing these findings, reduced absolute GLS has also been linked to myocardial microvascular dysfunction even in the absence of overt fibrosis, suggesting that impaired strain reflects early, preclinical myocardial injury in diabetic populations [78]. The above notions support the incorporation of GLS into routine echocardiographic evaluation for risk stratification in DBCM, facilitating earlier identification of high-risk patients who may benefit from closer surveillance or early therapeutic intervention. As such, GLS offers not only diagnostic but also prognostic utility, reinforcing its central role in the evolving echocardiographic assessment of diabetic myocardial involvement.

Beyond GLS, global circumferential strain (GCS) and global radial strain (GRS) provide complementary insights into myocardial deformation. While GLS typically declines first, GCS and GRS impairments become more evident as the disease progresses [75]. Additionally, segmental strain analysis using STE has revealed that the basal and mid-segments of the left ventricle are more affected in DBCM, which may play a role in diastolic dysfunction and increased myocardial stiffness [79].

Although strain imaging provides greater sensitivity than conventional imaging modalities its accuracy depends on image quality and remains affected by inter-vendor variability. While reference ranges for global longitudinal strain have been proposed, universally accepted cut-off values are still lacking which limits their broad clinical adoption.

### 4.2. Myocardial Work Index in DBCM

**Figure 4:**
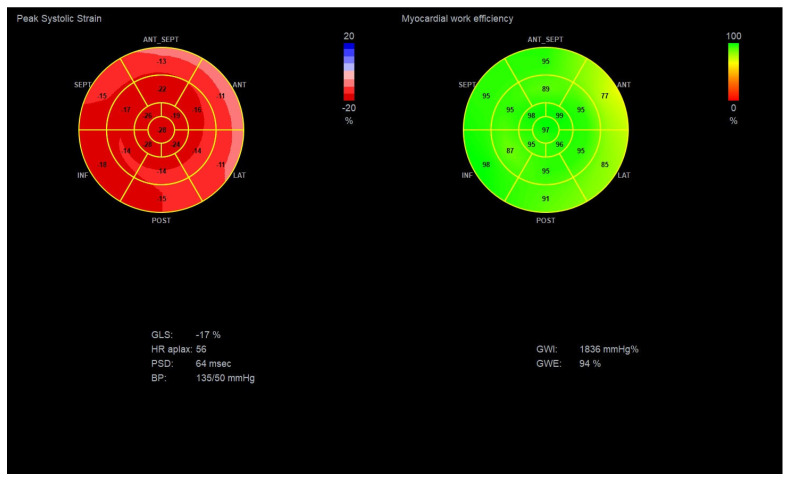
Global longitudinal strain and Myocardial Work Indices in a diabetic patient with suspected DBCM, LVEF: 55–60% and GLS: −17%. (Original images from authors’ institutions).

While strain imaging provides valuable insights into myocardial deformation, novel methods such as MWI offer a more load-independent approach to assess myocardial efficiency and contractile performance [76]. Derived from pressure-strain loops, MWI provides an evaluation of myocardial energetics, efficiency, and contractility. Key indices of MWI have been identified, including global myocardial work index (GWI), global constructive work (GCW), global wasted work (GWW), and myocardial work efficiency (GWE). All of them have shown significant alterations in the diabetic population [76]. Studies utilizing non-invasive MWI analysis have reported that diabetic patients exhibit reduced GWI and GCW, indicating impaired myocardial energy efficiency, while simultaneously there was an increase in GWW, suggesting heightened myocardial energy wastage [76]. These alterations in myocardial work are particularly pronounced in individuals with poor glycemic control, where HbA1c levels independently correlate with worsening myocardial efficiency [77]. Furthermore, longitudinal follow-up studies have indicated that MWI indices are predictive of future heart failure events, reinforcing their prognostic utility in DBCM [78]. The integration of myocardial work indices with strain imaging enhances the ability to detect subtle cardiac dysfunction at earlier disease stages. Notably, studies have demonstrated that MWI parameters outperform GLS in identifying subclinical myocardial dysfunction in diabetic patients with preserved LVEF, highlighting the potential of MWI for routine clinical application in this population [76] (Figure 4).

Nevertheless, myocardial work remains a relatively new technique requiring further validation in larger cohorts and its feasibility may be reduced in patients with suboptimal acoustic windows or arrhythmias.

### 4.3. Speckle Tracking Echocardiography of the Left Atrium

LA function plays a crucial role in maintaining cardiac hemodynamics, particularly in DBCM where diastolic dysfunction is a hallmark feature [80]. LA remodeling and dysfunction have been increasingly recognized as early markers of diabetic heart disease, where alterations in LA strain parameters show superior sensitivity than traditional volumetric assessment [81]. LA STE enables the assessment of three distinct phases of atrial function: reservoir, conduit, and contractile function. Among these, peak atrial longitudinal strain (PALS) and peak atrial contraction strain (PACS) have been identified as sensitive markers of subclinical diastolic dysfunction in diabetic patients [82]. Several studies have reported significantly reduced absolute PALS and PACS values in diabetic individuals compared to controls, with reductions correlating strongly with disease severity and glycemic burden [83].

LA stiffness, as assessed by LA strain imaging, has also been shown to be a strong predictor of elevated LVFP and progression to HFpEF in DM [84]. Additionally, LA strain abnormalities precede LA enlargement, suggesting that strain-based metrics may serve as an early warning system for identifying patients at risk of adverse cardiovascular outcomes [85].

**Figure 5:**
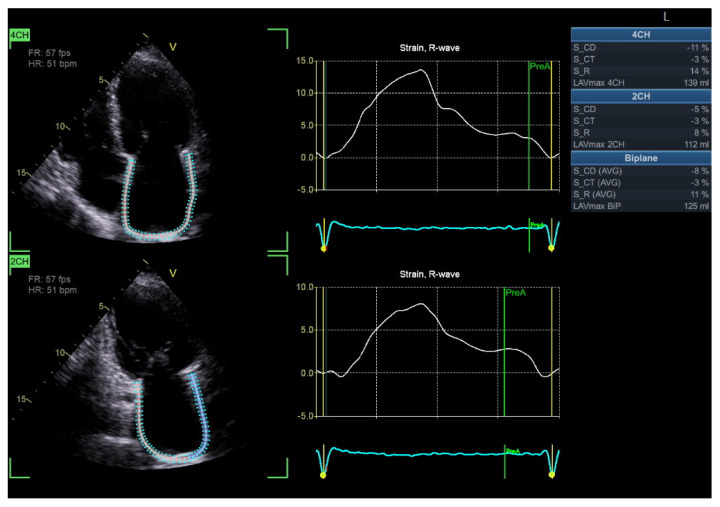
Impaired left atrial strain in a patient with DBCM (Original images from authors’ institutions).

Importantly, emerging data indicate that LA strain analysis may have independent prognostic value beyond conventional echocardiographic indices. A study by Swiatkiewicz et al. demonstrated that impaired LA strain was significantly associated with an increased risk of atrial fibrillation and adverse cardiovascular events in diabetic populations [86]. These findings suggest that LA strain assessment could be integrated into routine echocardiographic screening protocols for diabetic patients, particularly those with suspected diastolic dysfunction (Figure 5).

LA STE analysis is limited by rhythm and heart rate dependency and the lack of standardized reference values. Image quality is again a pre-requisite. The thin atrial wall increases susceptibility to noise, affecting its accuracy. In addition, abnormal septal motion, such as in high atrial loading conditions or interatrial septal aneurysm, may further impair the accuracy and reproducibility of measurements.

## 5. Stress Echocardiography

Unlike resting echocardiography, SE reveals impairment in systolic and diastolic function, providing an assay of increased LVFP—key pathophysiologic features of DBCM [87]. Following the subclinical changes, a reduced myocardial reserve—the heart’s capacity to increase function in response to stress- usually occurs long before the development of overt cardiac dysfunction at rest [88]. As the disease progresses, the heart develops impaired relaxation and increased stiffness, resulting in diastolic dysfunction. This is often the first detectable abnormality in dynamic testing [89].

### 5.1. Exercise Stress Echocardiography

Exercise stress echocardiography (ESE) is a valuable tool for unmasking diastolic dysfunction in patients with unexplained dyspnea and/or fatigue, particularly when the diagnosis remains uncertain due to inconclusive resting echocardiographic findings. The 2020 HFA–PEFF diagnostic algorithm recommends the use of ESE to confirm the diagnosis of HFpEF in cases of intermediate HFA-PEFF score [4,5,86]. The latter score is initially calculated from resting echocardiography parameters and natriuretic peptides levels. The addition of ESE is to reveal exaggerated elevations in LVFP and pulmonary artery systolic pressure (PASP) on exertion.

In clinical practice, ESE is commonly performed using a semi-supine ergocycle protocol with incrementally increasing workload (typically 25-watt increments every 2 min), enabling optimal image acquisition during each exercise stage [88,89,90]. Alternatively, upright bicycle and treadmill protocols are also used, particularly in centers without access to supine ergometry. However, these may limit real-time image acquisition, which can introduce variability due to rapid heart rate recovery [91].

**Figure 6:**
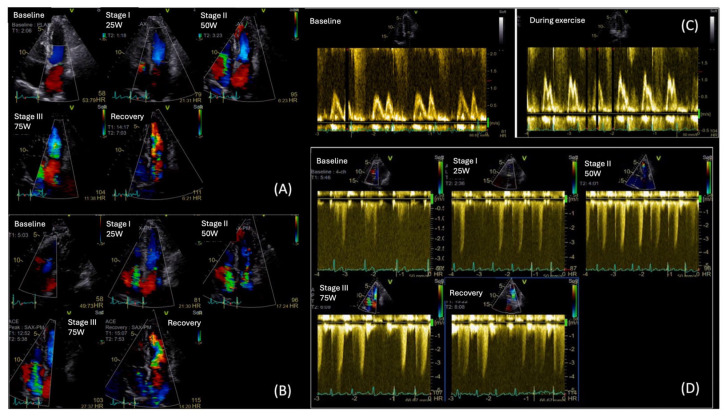
Diastolic Stress Echo in a T2DM patient with exertional dyspnea: (**A**) Color Flow Doppler (CFD) showing mild Mitral Regurgitation throughout the test, (**B**) CFD of Tricuspid Regurgitation (TR) augmenting during exercise, (**C**) Mitral inflow velocities at rest (left image) and during exercise (right image). We can observe a change in mitral inflow pattern, towards type II diastolic dysfunction, (**D**) Maximum TR velocity increase during exercise, from 2.5 m/s at rest to 4 m/s during exercise at low workload. (Original images from authors’ institutions).

During exercise, a rise in E/E′ and an increase in TR peak velocity (TRVpeak) are often observed in patients with HFpEF, reflecting an abnormal surge in LVFP and PASP, respectively. As heart rate rises, the impaired relaxation in diabetes leads to a disproportionate rise in LVFP and pulmonary pressures, exposing diastolic abnormalities not evident at rest [91,92,93]. Notably, according to the ESC consensus criteria, an exercise E/e′ ≥ 15 and/or TRVpeak > 3.4 m/s are indicative of HFpEF during stress [90]. Moreover, ESE may show an inadequate increase in stroke volume despite rising LVFP, differentiating cardiac limitations from non-cardiac causes of exercise intolerance (Figure 6).

While standard diastolic ESE remains the cornerstone in the evaluation of unexplained dyspnea in patients with suspected HFpEF, recent evidence suggests that a significant subset of symptomatic patients with negative ESE findings may still have HFpEF and a cardiopulmonary exercise test (CPET) could be useful. In a study by Verwerft et al., the use of exercise-induced pulmonary HTN, defined by an elevated mean pulmonary artery pressure to cardiac output slope (mPAP/CO slope > 3 mmHg/L/min), was shown to unmask abnormal cardiopulmonary reserve and impaired pulmonary vascular adaptation in patients with unexplained exertional dyspnea but normal ESE diastolic parameters [94]. Among these patients, elevated mPAP/CO slope strongly correlated with reduced peak VO_2_ and reduced ventilatory efficiency, supporting its diagnostic and prognostic utility in identifying HFpEF beyond classical criteria [94].

This approach is especially relevant in patients where DBCM may remain undetected by standard evaluation at its early stages. In the study by Gojevic et al., a higher percentage of diabetic patients with exertional dyspnea showed elevated mPAP/CO slopes during exercise compared to asymptomatic diabetic controls; however, the difference between groups was not statistically significant. This underscores the complexity of interpretation and highlights the need for further research to define the diagnostic and prognostic role of mPAP/CO slope in diabetic cardiomyopathy [95]. From the practical point of view, the combination of echocardiography and CPET has the potential for higher accuracy than ESE per se.

Beyond diastolic assessment, ESE evaluates contractile reserve, often impaired in the early stages of DBCM, even when the resting LVEF > 50%. A failure of exercise to increase LVEF or stroke volume may be an early sign of HFpEF [96]. In the diabetic population, GLS is often mildly reduced at rest and fails to augment appropriately during stress, indicating impaired systolic reserve [97]. Likewise, a blunted rise in peak systolic strain rate (SRs) during ESE reflects reduced contractile reserve, making these strain-based measures valuable for early diagnosis of subclinical DBCM [97].

MW provides an assessment of LV function and GLS by incorporating afterload assessment via the blood pressure measurement [98]. While MW has proven effective in identifying early, subtle abnormalities in HFpEF patients at rest, only a limited number of studies have investigated how these parameters respond to physiological stress. A dynamic evaluation may reveal impairment in these indices during stress that is masked at rest [98,99]. In the study of Zhang et al. (2025), which enrolled 40 patients with HFpEF and 40 normotensive controls, the HFpEF group demonstrated significantly lower increases in GWI and GCW, along with significantly greater increases in GWW and a more pronounced decline in GWE from rest to peak exercise compared to controls; these changes in MWI were closely associated with reduced exercise tolerance [100]. Inherent drawbacks of STE analysis in higher heart rates with reduced frame rates limit their clinical applicability [101].

### 5.2. Dobutamine Stress Echocardiography

Dobutamine stress echocardiography (DSE) is an alternative stress modality, particularly useful in patients unable to exercise or when ESE is inconclusive. In the context of DBCM, the role of DSE is limited to detecting only myocardial ischemia. Despite some scarce data, overall the DSE is unable to unmask cardiac diastolic abnormalities not evident at rest [101,102,103].

### 5.3. Coronary Flow Reserve in Stress Echocardiography

Coronary flow reserve (CFR), measured via stress echocardiography, has the potential to evaluate coronary microvascular function in the absence of obstructive epicardial CAD. CFR is typically assessed non-invasively using transthoracic Doppler echocardiography, by measuring coronary blood flow velocity in the distal left anterior descending artery (LAD) at rest and during maximal vasodilation, commonly induced with adenosine or dipyridamole infusion [104]. A ratio of hyperemic to baseline diastolic flow velocities reflects CFR, with values <2.0–2.5 indicating impaired microvascular function [105].

Its application provides insight into a pathophysiological feature of DBCM, which is coronary microvascular dysfunction (CMVD). Multiple studies have established the diagnostic and prognostic significance of CFR in the diabetic population. In a pivotal prospective study, a CFR cut-off value <2.5 in asymptomatic T2DM was strongly associated with MACE over a 79-month follow-up, confirming its value in early risk stratification [106].

More recent studies have proved the value of integrating multiple functional parameters—especially CFR, left ventricular contractile reserve (LVCR), and heart rate reserve (HRR)—into a single vasodilator stress echocardiography session for diabetic patients. In a study of 375 diabetic individuals, Cortigiani et al. introduced HRR in stress echocardiography as a prognostic index [107]. Among 636 diabetic patients with negative ischemia stress echocardiography, those with reduced CFR, LVCR, and HRR, exhibited the worst clinical outcomes, including greater all-cause and cardiovascular mortality, suggesting autonomic dysfunction as an additional pathophysiological contributor in DBCM [107].

ESE is highly valuable in detecting early myocardial dysfunction in diabetic patients. It carries important limitations including the need for experienced operators, challenging image acquisition at high heart rates and preload effects in clinical practice. GLS analysis during ESE is further constrained by loading conditions and lower frame rates at tachycardia, limiting its application in patients with suboptimal acoustic windows [14]. In comparison, dobutamine stress echocardiography may be used for coronary flow reserve assessment, however it is not recommended for the study of diastolic function.

## 6. Future Perspectives and Other Imaging Modalities

DBCM is frequently underdiagnosed among individuals with DM, yet its presence is associated with unfavorable clinical outcomes, such as death and heart failure [108]. Therefore, there is an urgent need for prompt diagnosis and accurate risk stratification of DBCM among the increasing diabetic population in order to improve their management and overall prognosis (i.e., intensification of antidiabetic treatment with proven cardiovascular benefits) [109]. Newer imaging techniques that allow for in vivo detection of early DBCM-related pathophysiological abnormalities can be used to complement echocardiography and enhance the assessment of this patient population [110].

Nuclear imaging techniques, such as single-photon emission computed tomography (SPECT) and PET, have been widely employed for metabolic and molecular imaging, opening new frontiers in the diagnostic and prognostic evaluation of patients with concomitant metabolic disorders and cardiovascular diseases, like DBCM [111]. The metabolic remodeling in DBCM has been extensively studied by means of PET, revealing significant alterations in myocardial substrate metabolism [111]. These changes occur even in the early adaptive/compensated phase of the disease, when echocardiographic findings may still be subtle [112]. Moreover, nuclear imaging enables the functional assessment of the autonomic nervous system, which plays a key role in the development and progression of HF [113]. Diabetes-related cardiac autonomic neuropathy (CAN) has been linked to major cardiovascular events and poor prognosis, suggesting that autonomic imaging might be useful in risk stratification and treatment monitoring of diabetic patients with HF [113,114,115]. However, inherent limitations of nuclear imaging (i.e., high cost, need for expertise, radiation burden with repeated tests) have restricted its use to research purposes or specific cases, raising questions about its role in clinical decision making of DBCM.

The DM-related perturbations in cellular metabolism lead to significant structural alterations in the myocardium over the course of the disease [3]. Echocardiography is the first-line imaging modality used to assess structural and functional abnormalities in DM patients [86]. However, CMR can more accurately assess the volume and the systolic function of both ventricles compared to echocardiography, which is an operator-dependent modality [116]. Besides that, CMR provides the additional benefit of comprehensive tissue characterization, revealing significant alterations in myocardial texture, with fibrosis being a notable characteristic of DBCM [37,117]. Late gadolinium enhancement (LGE) has been extensively used for the detection and quantification of fibrosis. Initially, fibrosis spreads diffusely throughout the myocardium, when LGE is not detectable. Alternatively, the CMR T1 mapping technique can non-invasively quantify diffuse fibrosis early in the course of the disease [118]. The extracellular volume fraction, a surrogate marker of diffuse fibrosis, has been closely related to glycosylated hemoglobin levels [119] and identified as an independent predictor of mortality among diabetic patients with diffuse fibrosis [120]. Later on, when large areas of myocardial cells are lost, focal (replacement) fibrosis develops [111]. The pattern of LGE distribution has been suggested to include basal lateral or basal inferolateral segments in diabetic patients [121]. Beyond volumetric assessment and tissue characterization, CMR also allows strain analysis through feature-tracking techniques, which can detect subtle impairments in myocardial deformation and complement echocardiographic strain imaging, particularly in patients with suboptimal echocardiographic images. These features not only enhance diagnostic accuracy but are also promising for follow-up of disease progression and therapeutic response [122,123]. However, the current data are very limited to draw specific CMR criteria for DBCM diagnosis. The presence of LGE among diabetic patients with nonischemic cardiomyopathy is associated with 3-fold higher risk for events [124]. Future large-scale studies are needed to elucidate the added value of CMR over echocardiography in DBCM diagnosis and prognosis, especially in patients with ambiguous echocardiographic findings.

More recently, artificial intelligence (AI) has emerged as a possible game changer in HFpEF. The latter remains difficult to diagnose due to its heterogeneity and subtle early manifestations, while DBCM is often underrecognized as a distinct phenotype [11,125]. In addition to supporting image acquisition, reducing operator dependence, and increasing reproducibility (i.e., automated strain analysis) AI can also harness large multimodal datasets to improve disease characterization. Machine learning based phenomapping has been shown to identify HFpEF subgroups with prognostic significance, uncover hidden patterns in imaging and biomarker data and even surpass traditional algorithms in predicting elevated filling pressures. Such approaches are valuable in DBCM, where early subclinical dysfunction may be missed by conventional indices [125]. In the future integrating AI derived echocardiographic markers with circulating biomarkers and electronic health records could enable more accurate risk prediction, refined patient stratification and personalized surveillance in diabetes. Although prospective validation is still needed, AI may transform DBCM from a diagnosis of exclusion to a condition that can be detected early and managed effectively.

DBCM is still an underdiagnosed condition, largely due to non-specific imaging criteria and a lack of clinical awareness. Echocardiography provides important diagnostic and prognostic information about DBCM and remains the cornerstone for its management [86] (Table 1). However, there are still some caveats in the diagnostic approach to DBCM. First, there is an eminent need for a standardized and unanimous definition of the disease, based on specific diagnostic criteria and thresholds derived from validated in real-world studies. Second, it is essential to define the phenotypic profile of patients who would benefit from echocardiographic screening for DBCM. Maintaining a high level of clinical suspicion is crucial not only for early diagnosis, but also for implementing a more rigorous surveillance in patients with initial normal echocardiographic findings. Finally, future studies should focus on establishing an optimal, multi-modality approach for the diagnosis, monitoring, and prognosis of DBCM, addressing the critical questions: who, when, and why.

## 7. Discussion

Despite recent advances in understanding DBCM, important uncertainties remain regarding its definition, diagnosis and clinical management. These challenges underscore the need for a more standardized and integrated approach.

Knowledge gaps persist at several levels. First of all, there is still no universally accepted definition of DBCM. While it is generally described as myocardial dysfunction occurring in diabetic patients in the absence of HTN and coronary artery disease, this definition is difficult to apply in real-world populations where comorbidities are common [3,11]. Secondly, diagnostic cut-offs for echocardiographic indices remain poorly validated. GLS, LA strain, and MWI, although sensitive markers of subclinical dysfunction lack universally accepted thresholds and along with their inter-vendor variability, their use in clinical practice becomes difficult [7,74,86]. Thirdly, most studies investigating echocardiographic parameters in DBCM are cross-sectional and relatively small, limiting the ability to establish temporal relationships between imaging abnormalities and clinical outcomes.

Several controversies also exist in the field of DBCM. There is a great debate on whether DBCM should be considered a distinct cardiomyopathy or one of the many phenotypes of HFpEF [13,20]. While DBCM shares features with HFpEF, including diastolic dysfunction and preserved ejection fraction in early stages, it also arises from diabetes-specific mechanisms such as metabolic derangements, microvascular dysfunction and insulin resistance [3,13]. This duality complicates both research design and clinical diagnosis. The prognostic value of novel echocardiographic indices also needs to be investigated. While impaired GLS and LA strain are consistently associated with adverse outcomes it is not yet established whether they add incremental value beyond standard measures or whether they can guide therapy [7,74,86].

These considerations highlight the need for prospective multicenter studies to validate advanced echocardiographic parameters and define robust cut-off values for the diagnosis and risk stratification of DBCM. Imaging algorithms that integrate conventional and advanced echocardiographic indices with biomarkers (i.e., natriuretic peptides, troponins, etc.) are needed. Furthermore, the role of stress echocardiography while promising for identifying latent diastolic dysfunction, remains underexplored in diabetic populations and requires further validation [14,87,95]. Recent technological advances (i.e., AI assisted echocardiography, automated strain analysis, and big data integration) offer the potential to improve reproducibility and support personalized care but their role in DBCM requires further validation [125].

**Figure 7:**
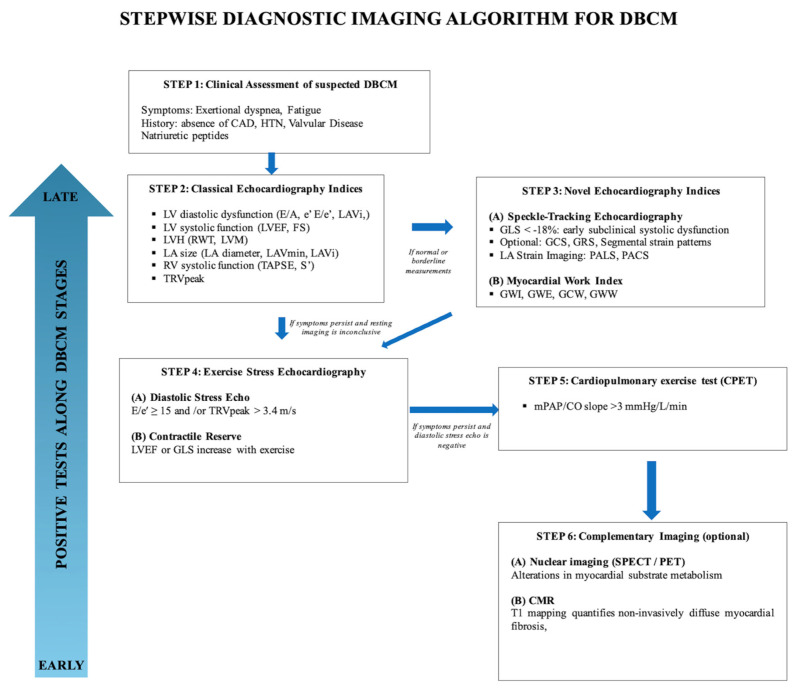
Stepwise diagnostic imaging algorithm for DBCM.

To address these gaps in this review we propose a stepwise multimodality imaging algorithm (Figure 7), which integrates conventional echocardiography, advanced echocardiographic indices, stress echocardiography and complementary imaging modalities to support the early diagnosis and risk stratification of DBCM.

## 8. Conclusions

DBCM is an often underrecognized complication of diabetes, associated with adverse cardiovascular outcomes and poor prognosis. Echocardiography, as a non-invasive and widely accessible imaging modality, remains the cornerstone for its diagnosis and risk stratification. Conventional indices such as LVH, systolic, and diastolic dysfunction are valuable but often fail to diagnose DBCM in its early, subclinical stages. Novel echocardiographic indices such as GLS, MWI, and left atrial strain offer greater sensitivity for early detection, with significant prognostic implications. In this context, ESE plays a pivotal role in unmasking exertional abnormalities in LVFP, myocardial reserve, and pulmonary pressures that are not evident at rest. DSTE is valuable in patients with unexplained dyspnea and normal values at rest, allowing for earlier identification of DBCM and guiding clinical decision making.

Despite advances in echocardiography, we lack standardized diagnostic criteria for DBCM, which is a significant challenge to early recognition and effective management of this entity. To support this, large prospective studies are required to confirm the diagnostic and prognostic value of advanced echocardiographic techniques and ESE, as well as to validate algorithms that integrate multiple imaging indices in diabetic populations.

## Figures and Tables

**Table 1:** Classical and novel echocardiographic indices of diabetic cardiomyopathy.

Variable	Indices	Diagnostic Challenges	Prognostic Value
LVH	RWT (PLAX), LVMI	Early changes may be subtle even in prediabetic stage; May be missed in obese, poorly imaged patients	Early feature of DBCM; Partially reversible with glucose control
LV systolic function	LVEF (Simpson)	LVEF may be normal at early stage; volumetric method better than 2D dimension assessment	Reversely associated with increased HF hospitalization, CV morbidity and mortality
	FS		
LA size	LA diameter, LAVmin, LAVi	LAVmin increases late; no cut-off value	LAVi as a predictor of CV morbidity and mortality in diabetic patients without known CV disease
Diastolic dysfunction	E/A, E/e’, e’ using TDI, LAVi, TRVpeak using Doppler	Influenced by age and preload; requires integration of multiple parameters	-Septal E/e’ ratio >15 in diabetic patients is associated with future HF and increased mortality.
			-Reduction in HbA1C levels may improve diastolic dysfunction, delaying the progression to HF
RV systolic function	TAPSE using M-mode, S’ using TDI	Affected by loading conditions; less standardized	Associated with increased CV morbidity and mortality
Speckle Tracking LV	GLS, GCS, GRS, segmental strain parameters	-Requires good image quality -GLS declines before GCS/GRS; segmental variations may confound interpretation; superior sensitivity than LVEF	-GLS is a stronger predictor of CV mortality than LVEF in diabetic patients -Optimization of DM treatment and reduction in HbA1C may improve GLS
Speckle tracking LA	PALS, PACS (reservoir, conduit, contraction)	Requires good image quality; dependent on HR and rhythm; validation is required	-PALS and PACS are sensitive markers of subclinical diastolic dysfunction in the diabetic population -Predictor of elevated LVFP and HFpEF progression in the diabetic population -Associated with an increased risk of CV events
Stress Echocardiography	E/E’ and TRVpeak during DSTE; less evidenced: Coronary flow reserve (CFR), LV contractile reserve, HR Reserve (HRR), mPAP/CO slope	DSTE has been incorporated in HFpEF algorithm; Requires equipment and expertise	Reduced CFR & HRR linked with MACE and mortality even in ischemia-negative stress tests

CFR, Coronary Flow Reserve; CV, cardiovascular; DBCM, Diabetic Cardiomyopathy; e’, early diastolic mitral annular velocity using tissue Doppler imaging; E/A, ratio of early to late transmitral diastolic flow velocities; E/e’, ratio of early transmitral flow velocity to early diastolic mitral annular velocity using tissue Doppler imaging; FS, Fractional Shortening; GCS, Global Circumferential Strain; GLS, Global Longitudinal Strain; GRS, Global Radial Strain; HbA1C, glycated hemoglobin; HF, Heart Failure; HFpEF, HF with preserved EF; HR, Heart Rate; HRR, HR reserve; LA, Left Atrium; LAVi, LA volume index; LAVmin, minimal LA volume; LV, left ventricle; LVEF, LV Ejection Fraction; LVFP, LV filling pressures; LVH, LV hypertrophy; LVMI, LV mass index; MACE, Major Adverse Cardiovascular Events; mPAP/CO slope, Mean pulmonary artery pressure to cardiac output slope; PACS, peak atrial contraction strain; PALS, peak atrial longitudinal strain; PLAX, parasternal long axis; RV, right ventricle; RWT, relative wall thickness; S’; peak systolic velocity of the tricuspid annulus (TDI); TAPSE, tricuspid annular plane systolic excursion; TDI, Tissue Doppler Imaging; TRVpeak, tricuspid regurgitation peak velocity.

## Data Availability

No new data were created or analyzed in this study.

## Abbreviations

The following abbreviations are used in this manuscript:

AGEs	Advanced glycation end products
BSA	Body surface area
CAD	Coronary artery disease
CAN	Cardiac autonomic neuropathy
CFR	Coronary flow reserve
CMD	Coronary microvascular dysfunction
CMR	Cardiac magnetic resonance
DBCM	Diabetic Cardiomyopathy
DM	Diabetes Mellitus
DSE	Dobutamine stress echocardiography
e’	Early diastolic mitral annular velocity using TDI
E/A	Ratio of early to late transmitral diastolic flow velocities
E/e’	Ratio of early transmitral flow velocity to early diastolic mitral annular velocity using tissue Doppler imaging
ESE	Exercise stress echocardiography
FA	Fatty acids
FS	Fractional shortening
GCS	Global circumferential strain
GCW	Global constructive work
GLS	Global longitudinal strain
GRS	Global radial strain
GWE	Global work efficiency
GWI	Global work index
GWW	Global wasted work
HbA1C	Glycated hemoglobin
HF	Heart failure
HFpEF	Heart failure with preserved ejection fraction
HRR	Heart rate reserve
HTN	Hypertension
IFG	Impaired fasting glucose
IGT	Impaired glucose tolerance
LA	Left atrium
LAD	Left anterior descending artery
LAVi	Left atrial volume index
LGE	Late Gadolinium enhancement
LV	Left ventricle
LVCR	Left ventricular contractile reserve
LVEF	Left ventricular ejection fraction
LVFP	Left ventricular filling pressures
LVH	Left ventricular hypertrophy
LVMi	Left ventricular mass index
MACE	Major adverse cardiovascular events
mPAP/CO	Mean pulmonary artery pressure to cardiac output slope
MW	Myocardial work
MWI	Myocardial work indices
PACS	Peak atrial contraction strain
PALS	Peak atrial longitudinal strain
PASP	Pulmonary artery systolic pressure
PET	Positron emission tomography
PKC	Protein kinase C
RAAS	Renin–angiotensin–aldosterone system
ROS	Reactive oxygen species
RV	Right ventricle
RVEDD	Right ventricular end-diastolic diameter
RWT	Relative wall thickness
S’	Peak systolic velocity of the tricuspid annulus (TDI)
SE	Stress echocardiography
SPECT	Single-photon emission computed tomography
STE	Speckle tracking echocardiography
TAPSE	Tricuspid annular plane systolic excursion
TDI	Tissue Doppler imaging
TR	Tricuspid regurgitation
TRVpeak	Tricuspid regurgitation peak velocity
